# Comparison of Two Common Outpatient Preparations for Colonoscopy in Children and Youth

**DOI:** 10.1155/2009/518932

**Published:** 2009-12-06

**Authors:** Carolina Jimenez-Rivera, Donna Haas, Margaret Boland, Janice L. Barkey, David R. Mack

**Affiliations:** ^1^Department of Pediatrics, Children's Hospital of Eastern Ontario, Ottawa, ON, Canada K1H 8L1; ^2^Children's Hospital of Eastern Ontario, University of Ottawa, Ottawa, ON, Canada K1H 8L1

## Abstract

Colonoscopies are often performed in children for diagnostic and therapeutic purposes. Our study compared two bowel-cleansing solutions: sodium picosulphate, magnesium oxide, and citric acid (Pico-Salax) with liquid magnesium citrate as preparations for colonoscopy. A retrospective chart review of all patients seen in the Gastroenterology outpatient clinic and who underwent bowel cleansing in preparation for colonoscopy from February to December 2006 was undertaken. Thirty-two children received Pico-Salax and 36 received liquid magnesium citrate. The tolerability of both solutions was similar. Most children in both groups had liquid stools and complete colonoscopies. Bowel preparation for a colonoscopy can be successfully achieved using either Pico-Salax or liquid magnesium citrate.

## 1. Introduction

Examination of the lower gastrointestinal tract by colonoscopy is often required in the pediatric age not only for diagnostic but also for therapeutic purposes [1]. There are situations when endoscopists are unable to perform complete colonoscopies due to inadequate bowel cleansing, which is particularly challenging for pediatric patients. Typically, children at our institution undergo a 2-day preparation, which includes the use of laxative agents (Pico-Salax or magnesium citrate) and a clear fluid diet. In some cases children undergo nasogastric tube lavage with a solution containing polyethylene glycol-electrolyte, however this requires a hospital admission which increases the cost of the procedure [2]. 

We compared two bowel-cleansing preparations in an outpatient setting with children undergoing colonoscopies; our primary outcome was to compare the efficacy of the cleansing for endoscopy. Secondary outcomes were to compare tolerability and stool consistency between the two groups. 

## 2. Materials and Methods

A retrospective chart review was completed for all patients seen in the Gastroenterology outpatient clinic who underwent bowel cleansing in preparation for a colonoscopy from February to December 2006 at the Children's Hospital of Eastern Ontario. 

The clinic nurses assigned each patient one of the two bowel preparations used in the outpatient clinic setting at their discretion. One preparation contained magnesium oxide, citric acid with sodium picosulphate (Pico-Salax, Ferring Pharmaceuticals Inc., Canada). This powder consists of sodium picosulphate 10 mg, magnesium oxide 3.5 g, and citric acid 12.0 g per sachet. Magnesium oxide and citric acid form magnesium citrate (when dissolved in water) and is administered as follows (per the manufacturer's recommendations): children from 1 to 6 years of age used 1/4 sachet, those from 6 to 12 years took 1/2 sachet and those from 12 to 18 years used 1 sachet of Pico-Salax once per day for two consecutive days. Liquid magnesium citrate was administered at a dose of 60 mL (1.74 g of magnesium citrate per 30 mL) in children between 10 to 15 kg, 90 mL in children between 16 and 20 kg, 150 mL in children between 21 to 35 kg, and 300 mL in children >36 kg also for two consecutive days. Additional measures included the administration of bisacodyl at a dose of 15 mg in children >20–35 kg or age 6–12 years of age and 20 mg in children >36 kg or 12–18 years of age following Pico-Salax or magnesium citrate. Castor oil (15–30 mL) was given to children younger than 6 years of age. Children remained on a clear liquid diet for the two days prior to the procedure.

Physicians recorded success of bowel preparation systematically including ease of endoscopy classified by need of irrigation and suctioning, minimal suctioning, or completion of procedure without irrigation and/or suctioning. Ease of endoscopy was considered excellent when there was minimal suctioning or there was no need of irrigation or suctioning. Tolerability was assessed by the recording of complaints such as vomiting, cramping and abdominal pain.

Statistical analyses were performed using R software (V2.7.2). Two-sided *P*-values of less than .05 were considered statistically significant. Continuous variables were summarized using mean and standard deviations. Categorical variables were summarized using frequency and percentage. Logistic regression was performed to compare the odds of primary outcome (excellent outcome) between the Pico-Salax and magnesium citrate groups with and without controlling for age. The rate of excellent outcome in children under age 6 and above age 6 was compared using Fisher's exact test. Secondary outcomes (tolerability and stool type) were also compared between the two groups using Fisher's exact test.

## 3. Results

A total of 68 children were included in the study, 32 received Pico-Salax and 36 received magnesium citrate. Mean age was 11.6 ± 3.9 years. Of the total of 68 candidates, 37 (54.4%) were males. Patient characteristics are reported in Table 1. 

Mean age was significantly different between the two groups (*P* = .0042). Only 3.1% (1/32) of children that received Pico-Salax were under age 6, whereas 22.2% (8/36) of children that received magnesium citrate were under age 6. Therefore, Pico-Salax was more likely to be used on older children whereas magnesium citrate was preferred for use on younger children. Fifty-nine percent (19/32) of children in the Pico-Salax group and 52.8% (19/36) of the magnesium citrate group had excellent outcome (OR = 1.31, 95% CI 0.5–3.4, *P* = .59) (Table 2). The logistic regression analysis controlling for age estimated that the odds ratio of having excellent outcome between the Pico-Salax group and magnesium citrate group was 1.50 (95% CI, 0.54–4.20, *P* = .44). Therefore, there was no statistically significant evidence that the odds of excellent outcome were different between the two bowel-cleansing solutions.

The percentage of excellent outcome between children less than 6 years of age and above 6 years was also compared. It was found that 55.6% (5/9) of children under age 6 had excellent outcome and 55.9% (33/59) of children above age 6 had excellent outcome (*P* = 1.00). Therefore, there was no statistically significant evidence that the percentage of excellent outcome was different between children less than 6 years and above age 6.

Tolerability of both cleansing agents was similar as evidenced by lack of difference in adverse events (vomiting, cramping, and pain), and most patients having no symptoms to report (Table 3). The magnesium group tended to have more liquid stools than the Pico-Salax group as reported in Table 3. However, this difference did not reach 0.05 significance level. There were more liquid stools in the magnesium citrate group compared to Pico-Salax, so there appears to be an offset of adverse events compared to firmness of stool.

The percentage of complete colonoscopy was 97% in the Pico-Salax group and 92.7% in the magnesium citrate group. There were two patients (one in each group) on whom complete colonoscopy could not be performed due to the presence of formed stools; these patients were eventually admitted to hospital for nasogastric tube lavage with Golitely (Polyethylene glycol 3350 and electrolyte solution). 

## 4. Discussion

Adequate bowel preparation is crucial for a successful colonoscopy. This can be significantly challenging in the pediatric population, as acceptance and tolerability of available agents might be poor. 

Two bowel preparation protocols are available at our center (Pico-Salax and liquid magnesium citrate), and used in combination with either bisacodyl or castor oil. Interestingly, there were no significant differences found between Pico-Salax and magnesium citrate in our study. Tolerabilility and efficacy of both methods were similar; this could be explained by the presence of magnesium citrate (an osmotic agent) in both solutions as the main ingredient. The addition of sodium picosulphate (a contact laxative that stimulates smooth muscle contraction) to magnesium oxide and citric acid (that forms magnesium citrate when dissolved in water) did not reveal higher benefits in our patient population. 

One of the limitations of the present study is potential imbalance in other clinical parameters (such as age) between the two groups. This imbalance could confound the effect of bowel preparation methods on the bowel preperation outcome. Young children are the most difficult to prepare for a colonoscopy. We compared outcomes in children less than 6 years to children above 6 years and found no difference between the ease of endoscopy or tolerability of the cleansing agent. This could be related to the small cohort in our study; 900 subjects would need to be included to detect a significant difference with 80% power. 

One would assume that older children's bowel preparation would be of better quality than younger children's, however this was not the case in our study. One could speculate that older children may not have been entirely compliant by taking the laxative agent or that the diet was not fully followed, as older children might have had less supervision.

There are controversies regarding the best combination of laxatives as well as the need for dietary restrictions. Abubakar et al. [3] stated that the use of oral bisacodyl for two consecutive days prior to the procedure and a phosphate enema the morning of the procedure provided excellent bowel preparation, these children did not follow any dietary restrictions. 

On the other hand, Dahshan et al. [4] concluded that the 2-day bisacodyl preparation yielded poor bowel cleansing as compared to either two Golytely or a combination of magnesium citrate and X-prep (senna fruit, sugar, and 7% alcohol).

Others believe that dietary restriction is a limiting factor for successful bowel cleansing and particularly in children. El-Baba et al. [5] showed that the administration of a prepackaged diet kit (low residue solid and liquid food kit) in combination with magnesium citrate and bisacodyl the day before the procedure was more effective than oral sodium phosphate and liquid diet in preparation for colonoscopy. 

Other methods of bowel cleansing, such as the oral administration of sodium phosphate solution proved a higher risk for electrolyte disturbances particularly hyperphosphatemia, hypocalcemia, and hypokalemia in up to 57% of adult patients [6, 7] and could be suspected to also be problematic in children and youth. 

Polyethylene glycol-electrolyte containing solutions are highly efficient in preparation for colonoscopy in children [4, 8], the limitation of this modality for bowel cleansing is the large volume required to achieve adequate results. Furthermore, most children are unable to take this solution orally and need to be admitted to hospital for nasogastric tube lavage, which can be inconvenient and more expensive.

Pico-Salax has been shown to have a high efficacy and safety in adults as a method of preparation for colonoscopies [9–13]. Pinfield and Stringer [14] described the efficacy of Pico-Salax in a pediatric randomized study that compared it to bisacodyl. All children (*N* = 32) on Pico-Salax were reported to have good or excellent preparation and fewer episodes of abdominal pain than in the group with bisacodyl administered. In our study, symptoms of abdominal pain or vomiting were low, with both groups having similar tolerability rates. 

The high efficacy of magnesium citrate as a cleansing agent in adults was reported by Chen et al. [15] where magnesium citrate in combination with bisacodyl was tolerated better and was more effective than castor oil. Magnesium citrate provided the day before the colonoscopy proved more efficacious than oral sodium phosphate in adults [16]. Most recently Sabri et al. [17] compared magnesium citrate and oral sodium phosphate showing similar tolerability and efficacy in adolescents. In the present study we used magnesium citrate in combination with bisacodyl or castor oil with good results that were similar to the use of Pico-Salax.

In summary, comparison of the two available bowel preparations showed no significant difference with regards to ease of endoscopy and tolerability. Colonoscopy with minimal or no need for irrigation and/or suctioning was achieved in about half of the patients on each group with the rest needing some intervention by the colonoscopist leading to achieving successful complete colonoscopy in the majority of patients. The retrospective nature of the study and the small study cohort represent potential limitations and larger studies may be warranted.

## Figures and Tables

**Table 1:** Patient characteristics.

	Overall	Pico-Salax	Magnesium citrate	*P* value
Total *N*	68	32	36	—
Age years, mean (SD)	11.63 (3.88)	13.01 (3.09)	10.40 (4.13)	.0042
Gender, *N* (%) of male	37 (54.4)	15 (46.9)	22 (61.1)	.35

**Table 2:** Comparison of ease of colonoscopy between Pico-Salax and magnesium citrate.

	Pico-Salax	Magnesium	Cruel odds ratio of ease of endoscopy (Pico-Salax/Magnesium)	95% CI for the cruel odds ratio	*P*-value
EO^†^ * N* (%)	19 (59.4)	19 (52.8)	1.31	0.50, 3.42	.59

^†^EO: Excellent outcome is minimal or no need for irrigation and/or suctioning.

**Table 3:** Comparison of tolerability and stool type between Pico-Salax and magnesium citrate.

	Pico-Salax (*N* = 32)	Magnesium citrate (*N* = 36)	*P*-value*
Adverse events^†^ (*N*, %)	1 (3.1)	4 (11.1)	.36
No symptoms	31 (96.9)	32 (88.9)	
Stool present			
Formed stool (hard, soft)	8 (25)	3 (8.3)	.098
Liquid	24 (75)	33 (91.7)	

*Fisher's exact test, ^†^Adverse events were vomiting, cramping, and abdominal pain.

## Appendix

See Tables 1, 2, and 3.
